# Houston County Medical Association

**Published:** 1887-06

**Authors:** 


### HOUSTON COUNTY MEDICAL SOCIETY.

A note from an esteemed friend and correspondent, Dr. A. D. Burroughs, of Lovelady, informs us of the organization (on the 14th of April), of the regular profession of Houston county, with a society, to be known as above. Dr. J. L. Lipscomb was elected President; Dr. Y. B. Smith, Vice-President, and Dr. J. L. Hall, Secretary. Meet quarterly at Crockett. Drs. W. C. Lipscomb and A. D. Burroughs were elected delegates to the State Association, but were prevented from attending, by professional business. Thus, one by one, the counties are wheeling into line. Not less than ten new county societies were enrolled at the recent meeting of the State Association.

A GENERAL INVITATION to the physicians of East Texas, is extended, to attend a meeting of the East Texas Medical Association, to be held at Lufkin, Angelina county, on 4th and 5th of July, prox., and become members.

We are pleased to note the growth and prosperity of this Association and to see "organization" thus progressing in East Texas.

